# Central Midwives Board for Scotland

**Published:** 1921-06-25

**Authors:** 


### Central Midwives Board for Scotland.

### PENAL CASES.

At a meeting of the Central Midwives Board for Scotland, Dr. J. Haig Ferguson in the chair, No. 1,606, Nurse Helen Miller, 41 Grove Street, Glasgow, was cited to answer charges of serious breaches of the Rules and failure to send for necessary medical assistance when urgently required, as also failure to notify cases of ophthalmia neonatorum. The Board found the charges to be proved, and instructed the Secretary to remove the name of Helen Miller from the Roll of Midwives, and to cancel her certificate.

No. 1,657, Nurse Mary Rennie McPherson Patton, 40 George Street, Aberdeen, appeared in answer to charges of having, on several occasions in maternity cases, in spite of warnings, used a dangerous drug without having noted in her Register of Cases the times and causes of its administration.

The Board found the charges to be proved, and expressed the opinion that the offences could not be adequately dealt with by censure or caution. It was decided to postpone sentence and to put the midwife on probation for three months for report from the Local Supervising Authority, and thereafter for a further period of three months for report from the Local Supervising Authority, and, in the event of an unfavourable report being received at the end of any of these terms, instructions were given that the name would forthwith be removed from the Roll and the certificate cancelled.

